# Discriminant Analysis as a Tool to Classify Grasslands Based on Near-Infrared Spectra

**DOI:** 10.3390/ani14182646

**Published:** 2024-09-12

**Authors:** Silvia Parrini, Maria Chiara Fabbri, Giovanni Argenti, Nicolina Staglianò, Carolina Pugliese, Riccardo Bozzi

**Affiliations:** Department of Agriculture, Food, Environment and Forestry (DAGRI), University of Florence, Piazzale delle Cascine 18, 50144 Florence, Italy

**Keywords:** forage resources, DAPC, herbage mass, FT-NIRS, grassland evolution

## Abstract

**Simple Summary:**

Knowledge of grassland system characteristics is of primary importance in livestock feeding to apply proper management strategies and maintain specific territories and their biodiversity. This study aims to test the application of discriminant analysis based on principal components to near-infrared spectra derived from intact fresh herbage. Samples were collected from recently sown (pure and mixed) grasslands and old meadows that were naturalized from the previously sown north-central Apennine (Italy). Classification achieved an overall assignment success rate of 77%, and the discrimination seemed to be applicable with success (up to 80%) for pure alfalfa crops and old permanent meadows derived from the same. Grass–legume mixtures and permanent meadows originating from old grass–legume mixtures achieved lower assignment success and seemed more similar. The application of discriminant analysis combined with near-infrared spectra is promising for grassland types, which differ in their botanical and chemical characteristics, for quick assessment of pasture qualitative levels, considering that it can be performed without drying and milling.

**Abstract:**

This study aims to classify plant communities by applying discriminant analysis based on principal components (DAPC) on near-infrared spectra (FT-NIRS) starting from fresh herbage samples. Grassland samples (*n*~156) belonged to (i) recent alfalfa pure crops (CAA), (ii) recent grass–legume mixtures (GLM), (iii) permanent meadows derived from old alfalfa stands that were re-colonized (PMA), and iv) permanent meadows originated from old grass–legume mixtures (PLM). Samples were scanned using FT-NIRS, and a multivariate exploration of the original spectra was performed using DAPC. The following two scenarios were proposed: (i) cross-validation, where all data were used for model training, and (ii) semi-external validation, where the group assignment was performed without samples of the training set. The first two components explained 98% of the total variability. The DAPC model resulted in an overall assignment success rate of 77%, and, from cross-validation, it emerged that it was possible to assign the CAA and PMA to their group with more than of 80% of success, which were different in botanical and chemical composition. In comparison, GLM and PLM obtained lower success of assignment (~52%). External validation suggested similarity between PLM and GLM groups (93%) and between GLM and PLM (77%). However, a dataset increase could improve group differentiation.

## 1. Introduction

Grasslands are ecosystems or ecological communities representing almost a third of the earth’s surface and 22% of European land area [1,2], involving high biodiversity [3], to which biotic and abiotic factors are linked. In this context, pastoral lands are indirectly associated with maintaining biosphere stability, and nowadays, they have a significant role in the mitigation strategy for climate goals [1]. In the last decades, the grassland area has decreased because of other agricultural uses, such as conversion to arable land [2,3], to support the continuously increasing food demand. Land use intensification or changes in agricultural land use and other anthropogenic activities, such as intensive livestock grazing, are the principal drivers of biodiversity loss. This reduces local species diversity and biotic homogenization at larger spatial scales [4,5]. The importance of grasslands in the world for livestock systems is recognized by their distribution, occupying 3.5 billion ha, of which almost 2 billion ha are used for grazing livestock, even if not all grasslands are grazed by domesticated animals. Knowledge of the grassland system characteristics is the basis for both the conservation of biodiversity and the improvement in grassland management practices, according to [6], who reported that the priority of information availability on pasture characteristics contributes to achieving optimal pasture management, particularly if devoted to animal grazing.

Studies [7] have reported the use of hyperspectral images (HSIs) to detect and classify grassland botanical composition, especially crops and weeds, implying high use of human and material resources and the combination of hyperspectral images with remote sensing [8,9,10,11]. The use of remote sensing involving spectral bands and structural (textural, geometric) features has shown that similarities in grassland characteristics confound their classification [12]. Other research [13] performed, with a laboratory-scale NIR-HSI system, the discrimination of grassland species of three botanical functional groups as follows: Poaceae, Fabaceae, and species belonging to other families focusing on the ecological plant aspects.

Near-infrared spectroscopy (NIRS), considering the full range of near-infrared regions, could represent an opportunity to discriminate grassland features for the livestock sector. The advantage of NIRS is that the methodology is speedy and non-destructive, has a high throughput, and can be cost-effective. On the other hand, NIRS technologies also include implications such as the need for extensive calibration of the prediction equations and the lack of cost-effectiveness in combining external/commercial calibration. Near spectroscopy has already been applied directly to estimate the chemical composition of natural or artificial fresh samples of mixed and pure herbage [14,15,16]. At the same time, fewer studies have been performed on the classification of vegetation typologies in a livestock direction. Study [17] tried to develop NIRS models to predict species composition in mixed swards, considering mainly artificial samples. Classification using a qualitative discriminant procedure of fresh grassland vegetation by spectral information (NIRS), based on different functional traits [18], can save labor in research and quickly evaluate grassland classes. The recognition of specific species or botanical groups, as well as different economic levels of grassland, in the long term, could be of topic appeal in the online application on the field.

The present study aims to test the application of discriminant analysis based on principal components to near-infrared spectra derived from fresh herbage samples originating from naturalized and sown grasslands in north-central Apennine (Italy). The hypothesis assumes that distinct plant communities can be classified by near-infrared spectra capturing and evidencing information by a discriminant analysis based on principal components.

## 2. Materials and Methods

### 2.1. Forage Sample Set

**Figure 1:**
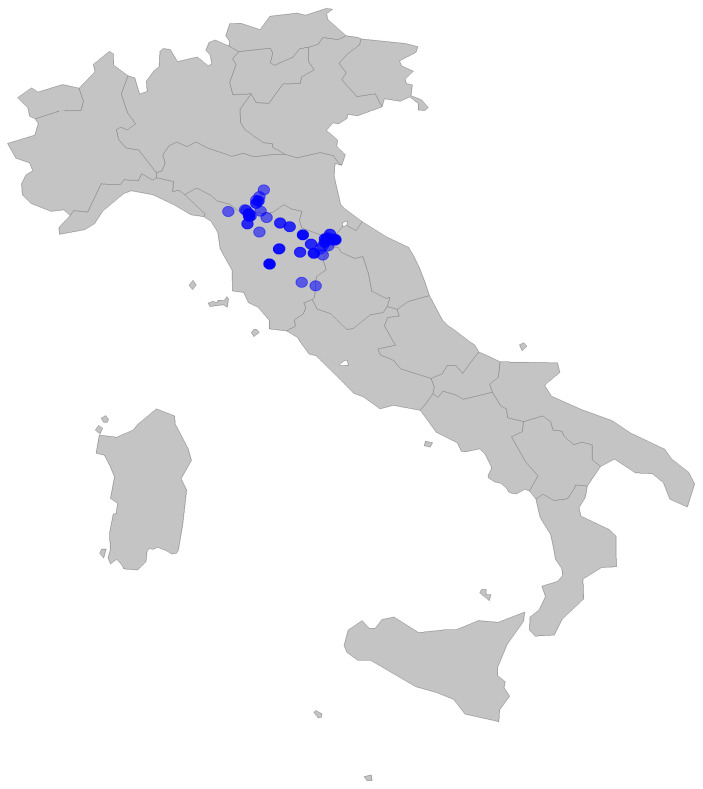
Sampling sites.

Samples were collected in different areas of north-central Apennine (Italy) at an altitude higher than 300 m.a.s.l. They are referred to the provinces of Modena, Bologna, Pistoia, Prato, Firenze, Arezzo, and Siena, which were representative of the grassland types in the study area. Samples were collected from a 1 m^2^ representative area at each site [19]. The sample sites are reported in Figure 1. Sampling was repeated 1 to 3 times for each site, covering different phenological phases along the vegetative season. Sampling was performed from 2016 to 2022. Forage sampling refers to cropped and re-colonized grasslands devoted to hay production and animal grazing, as reported by Parrini et al. [16].

For each sample, information on age (from sowing to sampling), present type, original crop, and composition was collected. Grassland species were identified (Table 1) using a tray with a white background. The weight and proportion of each botanical group (legumes, grasses, and other forbs) for each sample were identified [20,21] and expressed as the percentage contribution to the herbage mass on a wet matter basis.

Subsequently, the samples were classified into the following four groups using criteria based on their origins and ages (summarized in Table 2):

CAA (*n* = 27): *Alfalfa* crops recently established (less than 4 years old), presenting a very high percentage of sown legumes (~80%) mainly identified as *Medicago sativa*, grasses belonging to the genera *Bromus, Lolium,* and *Poa* (~16%), and other forbs, representing the remaining 4%.

GLM (*n* = 33): grass–legume mixtures recently established (less than 5 years of age). These presented a high presence of legume species (~60%), mainly represented by species belonging to the genera *Medicago*, *Lotus*, *Onobrychis*, and *Trifolium*, grasses (~35%) belonging to the genera *Dactylis*, *Festuca*, *Lolium, Poa*, and *Phleum*, and other forbs (~5%).

PLM (*n* = 36): permanent meadows originated from old grass–legume mixtures (more than 7 years old) re-colonized by native species, mainly grasses (~55%) belonging to the genera *Dactylis*, *Festuca*, *Phleum*, *Avena*, and *Bromus*, low legume presences (~31%), mostly *Lotus* and *Trifolium*, and other forbs (about 14%).

PMA (*n* = 60): permanent meadows derived from old alfalfa meadows (over 7 years old) re-colonized primarily by grasses with some forbs. These permanent meadows were highly naturalized by local species belonging to grasses (~62%), such as *Avena, Bromus*, and *Hordeum*. In comparison, the ground cover of legumes was reduced to 26% (mainly *Trifolium*) and other forbs (about ~12%).

### 2.2. Sample Preparation, Spectral Measurement, and Chemical Analysis

Each sample of fresh herbage was reduced by hand-clipping to 2–4 cm diameter. Three aliquots were randomly selected and scanned for each sample by a cup spinner after acquiring a background spectrum. Each spectral measurement was obtained from 32 scans performed at a wave number resolution of 4 cm^-1^ over the range of 1000 to 2500 nm wavelength (4000–9999 cm^−1^ wavenumber) and corrected against the background spectra of the room environment. Absorbance data were collected as log 1/R, and 3112 data points were recorded per sample by FT-NIRS Antaris II model (Thermo Scientific, Waltham, MA, USA), maintaining a constant temperature in the laboratory room. An average spectrum of the three measurements was calculated for each sample.

After the FT-NIRS collection, the samples were dried in a forced-air oven to constant weight and ground through a mill (Brabender OHG, Duisburg, Germany) to pass 1 mm. The chemical analysis was performed according to the following AOAC [20] methods: dry matter (DM) content using the 934.01 method, crude protein (CP) by the 976.05 method, ash via the 942.05 procedure, crude fat (EE) using the 2003.05 method, acid detergent fiber inclusive of residual ash (ADF), and acid detergent lignin (ADL) using the 973.18 method. Neutral detergent fiber, including residual ash (NDF), was determined according to the procedure described by [21].

### 2.3. Statistics Analysis and Chemometrics Application

Individual spectra were evaluated, even if no pre-treatment was used, and all wavelengths were equally weighted. Outliers were detected based on the high residual values and high Hotelling’s T2 statistic values calculated from the sample scores, which were indicated as the variation in each sample within the model. Missing and abnormal values were discarded from the dataset.

Principal components analysis (PCA) was conducted using the “prcomp” function of the R package [22] on the absorbance of the 3112 wavenumbers to observe the differences among groups.

Discriminant analysis of principal components (DAPC) was performed on spectra using the adegenet R package [23], considering the four origin groups as a classification factor. The discriminant analysis of principal components approach was applied to identify the potential cluster separation of NIR spectra based on the four grassland groups previously described (CAA, GLM, PMA, PML). The aim was to evaluate the spectra assignment to the own group.

DAPC was performed in two steps as follows: first, a PCA was conducted and then, a small number of selected PCs (instead of 3112 wavenumbers) was used as input for the linear discriminant analysis (LDA). The number of PCs subsequently used for LDA was selected by cross-validation (CV), where the data were split into training (TRN) and validation (VAL), represented respectively by 70% and 30% of the total dataset. The procedure for selecting PCs considered that the number of replicates was set to 30 for each level of PC retention, the maximum number of tested PCs was 20, and the number of PCs retained was established on the highest mean success.

For spectra discrimination, two approaches were applied as follows:

- Cross-validation, where the full dataset was considered simultaneously using all data for model training. Subsequently, discriminant functions were extracted based on all samples, and the correct assignment of samples to their origin was assessed.
- A semi-external validation, in which the assignment group was tested in the training set without any samples belonging to the group. Therefore, spectra of the testing sample consisting of one group were classified into the remaining 3 groups of the training set, showing the similarity among groups.

Sample classification was explained by the percentage of successful assignments. The outcomes were reported in a confusion matrix, with rows representing the classes to which samples belonged and columns representing the classes to which samples were assigned by the classification rule [24].

## 3. Results

Descriptive statistics of chemical analysis are reported in Table 3, considering class based on grassland type.

In the full dataset, five samples were considered outliers based on the high Hotelling’s T2 statistic values of the wavelengths and removed from the following analysis. In contrast, one sample was removed for chemical analysis after data quality control was performed. The cultivated meadow groups (CAA and GLM), characterized by a higher presence of legume forages, especially alfalfa, showed high protein contents and lower levels of dry matter and NDF. Conversely, naturalized meadows (PMA and PLM), as expected, showed a lower CP content and a higher DM and NDF content.

**Figure 2:**
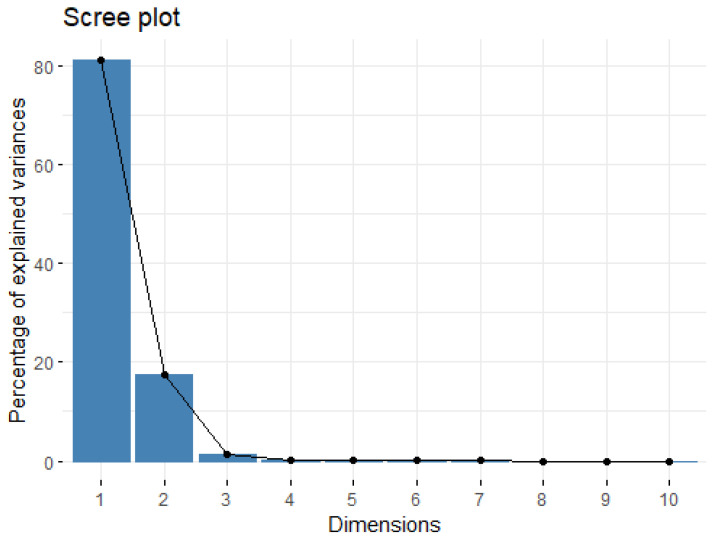
Scree plot of the total explained variance of the spectra data.

Regarding the PCA, the total explained variance reported in the scree plot (Figure 2) presented 10 dimensions per newly defined principal component on the x-axis and the percentage of the explained variance on the y-axis (based on eigenvalue). The first PC dimension represented 80.9% of the variance; the second represented 17.3%, and the third represented 1.3% of the explained variance, with little information gained by adding other PCs. Indeed, most of the spectra variability (roughly 98%) was summarized by the two principal components, and the number of principal components to be retained could not exceed three dimensions for the explorative PCA analysis. The explorative PCA analysis (Figure S1) positioned each cluster on PC1 and PC2 in the two-dimensional scatter plot of scores. PC2 allowed the discrimination of the PMA fields from the others, while PC1 could not cluster the group mentioned above; otherwise, PC1 slightly separated PLM from others.

**Figure 3:**
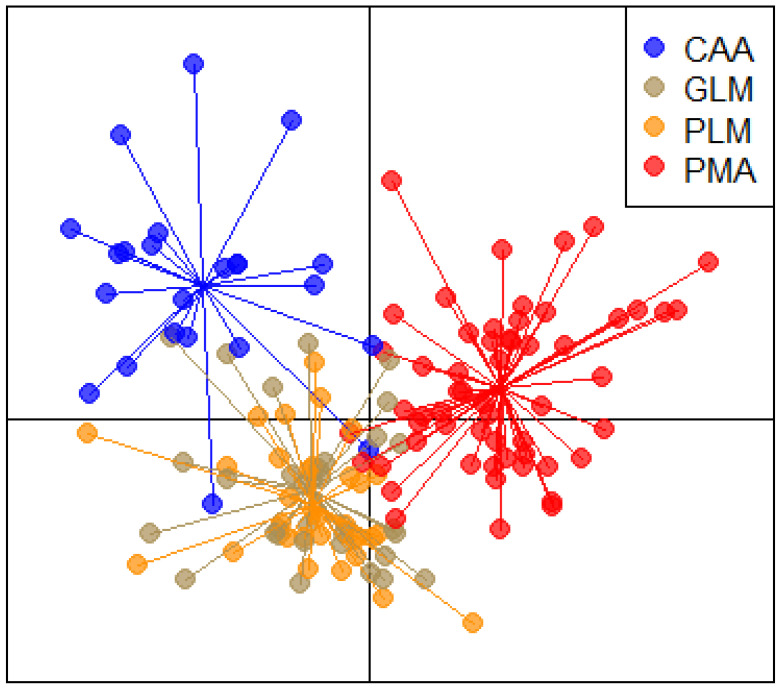
Biplot of the DAPC analysis.

The DAPC analysis results are shown in Figure 3, considering the full dataset. This approach defined three groups based on the diversity of the spectra as follows: (i) CAA, (ii) PMA, and, finally, (iii) PLM and GLM resulting overlaps. The first 11 PCs were used in the final DAPC model, considering that the root mean square error decreased from 0.64 obtained by PC1 to 0.31 by PC11. The overall assignment success rate for the origin group was 76.9%. Using three discriminant functions, this model retained the lowest root mean square error and explained about 99% of the total variance.

Table 4 reports the post-successful assignment of individuals to their original clusters based on the previous model in cross-validation as a confusion matrix. The values higher than 80% of correct assignment for CAA and PAM suggest clear-cut clusters, while GLM and PLM were confirmed as admixed, attributing 30% and 37% of samples to each other.

The results of the semi-external validation approach are presented in Table 5 as percentages of posterior assignment probability. The training set did not include the validation data set for each group. Indeed, the membership of a sample to its class could not be evaluated; contrariwise, it was possible to highlight the similarity among groups.

The following two groups showed a high percentage of assignment to only one group: PLM with GLM (93%) and GLM with PLM (77%), showing reciprocity between the pairs. Also, CAA was assigned 56% of the time to GLM and in a similar percentage to PLM and PMA (~21%). Samples of the last group, PMA, were mainly assigned to PLM (52%) and GLM (22%), and a small group (8.8%) were assigned to CAA. Even if there was no complete reciprocity in the pair’s assignment, it is evident that GLM was the group that reported the highest percentage of assignments, and CAA was the lowest.

## 4. Discussion

A fast assessment of grassland system characteristics is the basis for improving biodiversity conservation, applying appropriate management practices, and evaluating grassland resources in livestock systems, mainly if samples can be analyzed without drying time. Some previous studies [25,26] reported that grassland ecosystems, including sown and natural meadows, are challenging to investigate because of their natural diversity and complexity, as well as the wide range of management and environmental conditions that they are exposed to, even if monitoring both quality and origin features are primary for livestock management purposes [27].

The chemical composition of the grassland samples studied suggested that the qualitative characteristics of the grassland were directly affected by the presence of species and their abundance in the processed samples. In particular, recent legume crops (CAA and GLM) were favorably linked with increasing protein and fat content [28,29,30], even if it seemed that there was no direct association with the content of fiber, probably covered by the effect of the entire vegetative season included in the sampling. Previous research in different Italian regions, i.e., Emilia–Romagna [31] and Calabria [32], reported a lower crude protein content for prevailing alfalfa crops and permanent meadow hay compared with the results obtained in our study. However, beyond species composition, many factors can affect the nutritional value of herbage, i.e., climatic conditions, soil composition, phenological state [25], nutrient availability [33], and stage of maturity [34,35].

The present study showed that the explorative PCA on spectral data could represent the main part of variability using a low number of PCs (two components retained, explaining 98% of the total variability). The low number of PCs used confirmed the direct association among plant tissue, their structure, and wavelength characteristics, according to Asner [36], who described this phenomenon as the wavelength dependence of plant tissue. It is important to point out that spectral data were used as raw spectra derived from fresh herbage without applying preprocessing treatments on the samples and data. A study [18] suggested using raw spectral data without preprocessing steps because they did not decrease performance but could complicate the models. In quantitative NIRS calibration models, a reduced number of math pre-treatments on spectra is often suggested to avoid the complexity of interpretation and artifacts derived from the loss of some information and the minor structural differences among very similar signals [24]. Still, in the chemometrics approach, it is also interesting to note that our study used the full range of near-infrared regions for the classification, and it was not necessary to provide different weights or select some regions. Such considerations are concordant when there is no evident wavelength-specific relevance and the model does not suggest a prior variable region that implies a variable selection based on the absence of differences in the variance explained by the model [37].

The results of DAPC coupled with FT-NIR pointed out an overall assignment of 77% to its origin group, highlighting two clear trends as follows: PMA and CAA seemed to be the clusters more distant from the others. At the same time, GLM and PLM were almost overlapped, indicating that the spectral information contained in both types of spectra was very similar. From the first scenario of DAPC analysis (cross-validation), it emerged that it is possible to correctly assign and classify two types of grassland (CAA and PMA) extreme for their botanical and chemical composition, even if they originated from the same crops. Alfalfa species effectively characterized the first group of samples from which PMA differed concretely from the prevalence of Gramineae, which is acknowledged to encroach remarkably on pure legume fields after the average persistence of the crop [38]. In accordance with our research, ref. [31] reported the highest assignment rate for alfalfa hay, explained by the different anatomical structure and subsequent proportions in terms of leaves and fibrous stems of alfalfa legume compared with others [39], although the study discriminated based on chemical characteristics and not on spectral data.

In our study, the chemical composition of the sample groups was translated into a high and low protein content, different dry matter content, and NDF composition. Indeed, botanical composition was the factor that affected the chemical characteristics (consistent with previous research, e.g., [39]) and, in turn, the structure of the spectra in the near-infrared regions. At this time, it is known that NIR regions are represented by the absorption produced by the combination of harmonics and overtones of the fundamental frequencies of functional groups. However, the high water content of fresh herbage can modify and complicate results. A previous study [7], using a NIR-HSI system in the region between 1100 and 2400 nm, reported better discrimination of fresh herbage species according to botanical family membership, with results including between 96 and 100% classifications of Gramineae, the Leguminosae family (Faba), and other botanical families (OBF) using the PLS-DA algorithm. Nevertheless, those authors reported that the results worsened if the number of grassland species increased in a group. Our study did not confirm their results because the PMA samples obtained better results but comprised the most significant number of species. However, the model selected can be an affecting factor. For example, better results were obtained using multilayer perceptron (MLP) than PLS-DA in species group identification by HIS technology considering grasses, legumes, and herbs by [18]. However, in comparison with our study, those researchers used a higher number of samples and a reduced site area: two sites in [18] and one area typology in [7].

The posterior assignment in a semi-external validation scenario is focused on the similarity between the groups, allowing the separation of two trends. First, the reciprocal relationship between PLM and GLM (93%) and GLM and PLM (77%) was confirmed, indicating that the spectral information included in both types of spectra was very similar, as overlapped in Figure 3. The status of GLM and PLM, which had only different ages, suggest that spectra similarity is probably related to lower differences in chemical composition. On the contrary, the CAA and PMA assignments showed more significant variability in the assignment’s success. However, reasonably, the spectra of CAA were closer to GLM, and PMA was mainly assigned to PLM. The results of our study suggested that the greater diversity of information derived from the spectra allowed the assignment to be the most successful. Accordingly, study [17] reported the positivity of high variation present in the absorption of spectral data because of calibration performance when a real sample model was developed compared with artificial samples and spectra, even though the botanical composition was the same. The group that collected the highest similarity success from other groups was GLM, probably because it represented both Gramineae and Leguminosae in intermediate degree and prevalence. However, as expected, pairs in the main case were not reciprocal among the assignment success, as already verified by [31] in the discrimination of hay based on chemical composition.

The application of DAPC and NIR spectra on fresh herbage, not dried or ground, could be helpful as quick support in the field technicians for CAA and PMA, achieving more than 80% of assignments, according to previous studies [18,31], especially for prevalent legumes. The discrimination performance should be improved for specific grasslands as GLM and PLM, which are closer in botanical and chemical composition than CAA and PMA. One step, which is often used in chemometrics, could be increasing the number of samples for the specific type, focusing on the spectra contribution of variation in the dataset. Finally, in the long term, applying research systems to practical use could be the first step to enable grassland management in a more sustainable mode, adapting agriculture systems to environmental change according to the new suggestion of FAO [2]. In addition to ecological aspects, the output of such a study will include the recognition of economic values based on distinct grassland classes. The perspective of future studies should include synergic cooperation among agronomists, animal experts, and engineers for the construction of devices that will be low-cost, suitable for users, and friendly and speedy for different stakeholders. In the meantime, it is necessary to consider that technologies need a long time to achieve accessible interfaces that are compatible and comprehensible for direct use by producers in the field. In this context, both policy and efficient strategy information will be useful [40].

## 5. Conclusions

Considering the successful assignment of specific grassland types to their groups, their sensitivities, and their lower similarities with other groups, the newly developed DAPC method coupled with NIR could effectively differentiate and classify specific grassland types based on fresh herbage directly. Noteworthily, the top successful assignments were achieved by recent pure alfalfa (CAA) and permanent meadows derived from the previous meadows (PMA) that were reciprocally extreme in chemical composition and botanical prevalence, even if they originated from the same crop. Effectively, the greater diversity of information derived from the spectra allowed the assignment to be the most successful. Further steps should be undertaken to increase the dataset to improve differentiation between grass–legume mixtures and permanent meadows with intermediate composition, improve the accuracy and precision of the model, and, in the future perspective, develop the dataset for identifying toxic or invasive species.

The fast classification of fresh grassland herbage and derived hay could be useful as a practice to improve grassland management and evaluate the value of production properly. In the future perspective, it could even be carried out without drying and grinding as a basis for developing models on nutritional value. From an ecological point of view, NIRS coupled with DAPC can be applied to the study of site-specific management and possible future applications for the study of grassland evolution.

## Figures and Tables

**Table 1:** Grassland species identified in the samples of the present study.

Grasses	Legumes	Other Forbs
*Arrhenatherum elatius* L. *Agropyron repens* L. *Agrostis tenuis* L. *Avena sativa* L. *Arrhenatherum elatius* L. *Bromus erectus* Huds. *Bromus hordeaceus* L. *Bromus sterilis* L. *Cynosurus cristatus* L. *Dactlis glomerata* L. *Festuca arundinacea* Schreb. *Hordeum murinum* L. *Hordeum vulgare* L. *Lolilum multiflorum* Lam, *Lolium perenne* L. *Poa pratensis* L. *Poa trivialis* L. *Phleum pratense* L.	*Medicago sativa* L. *Lotus corniculatus* L. *Hedysarum coronarium* L. *Onobrychis viciifolia* Scop. *Trifolium pratense* L. *Trifolium repens* L.	*Achillea millefolium* L. *Amaranthus retroflexus* L. *Angelica archangelica* L. *Artemisia vulgaris* L. *Bellis perennis* L. *Brassica napus* L. *Capsella bursa-pastoris* L. *Cerastium arvense* L. *Cichorium intybus* L. *Convolvulus arvensis* L. *Dianthus carthusianorum* L. *Erigeron annuus* L. *Geranium dissectum* L. *Geum urbanum* L. *Hypericum perforatum* L. *Lamium purpureum* L. *Leucanthemum vulgare* Lam. *Malva sylvestris* L. *Mentha arvensis* L. *Myosostis arvensis* L. *Ornithogalum umbellatum* L. *Papaver rhoeas* L. *Picris hieracioides* L. *Potentilla erecta* L. *Plantago lanceolata* L. *Potentilla erecta* L. *Ranunculus arvensis* L. *Rumex obtusifolius* L. *Senecio vulgaris* L. *Silene vulgaris Moench* *Taraxacum officinale* F.H. Wigg. *Tragopogon* spp. L. *Verbena officinalis* L. *Veronica persica* Poir

**Table 2:** Summary of forage characteristics by classification groups.

	CAA	GLM	PLM	PMA
Number of samples	27	33	36	60
Grassland type	alfalfa crops	grass–legume mixtures	permanent meadows naturalized	permanent meadows naturalized
Age (years)	1–4	1–5	7–12	7–12
Original crop	alfalfa crops	grass–legume mixtures	grass–legume mixtures	alfalfa crops
Legumes % (µ, ± SD)	80 ± 15	60 ± 19	31± 26	26 ± 18
Grasses % (µ, ± SD)	16 ± 10	35 ± 16	55 ± 27	62 ± 17
Other forbs % (µ, ± SD)	4 ± 0.8	5 ± 1	14 ± 3	12 ± 5

CAA: pure alfalfa crops; GLM: grass–legume mixtures recently established; PLM: permanent meadows originated from old grass–legume mixtures; PMA: permanent meadows originated by old alfalfa.

**Table 3:** Descriptive statistics of the chemical analysis of the samples.

	DM %			CP (g100g^−1^)			Ash (g100g^−1^)			EE (g100g^−1^)			NDF (g100g^−1^)			ADF (g100g^−1^)			Lignin (g100g^−1^)		
	Mean	SD	Range	Mean	SD	Range	Mean	SD	Range	Mean	SD	Range	Mean	SD	Range	Mean	SD	Range	Mean	SD	Range
CAA	14.7	3.1	11.5–21.1	21.0	2.9	15.5–25.0	11.6	1.5	8.3–13.6	2.4	0.5	1.6–3.4	48.9	8.0	37.2- 72.9	36.6	4.5	25.6–43.7	7.5	2.6	1.9–9.7
GLM	16.8	3.1	12.2–25.3	20.4	3.1	14.6–25.8	11.0	1.5	8.1–13.2	2.5	0.5	1.4–3.7	45.7	7.7	30.8- 65.6	33.4	6.2	21.3–45.5	6.8	2.1	2.7–9.6
PMA	25.2	8.4	11.1–43.9	15.2	4.5	7.4–24.1	10.0	1.8	4.5–12.5	2.2	0.6	0.6–4.0	54.5	8.7	33.0–71.3	36.2	7.1	12.5–48.8	7.0	1.9	2.3–9.9
PLM	20.5	6.7	12.4–43.0	16.9	4.9	8.9–24.8	10.3	1.8	6.5–11.9	2.3	0.6	1.0–3.8	51.1	10.7	32.8- 71.7	35.5	6.9	24.9–50.1	6.4	2.3	2.5–10.1

CAA: pure alfalfa crops; GLM: grass–legume mixtures recently established; PMA: permanent meadows originated by old alfalfa; PLM: permanent meadows originated from old grass–legume mixtures. DM: dry matter; CP: crude protein; EE: crude fat; NDF: neutral detergent fiber; ADF: acid detergent fiber; ADF: acid detergent fiber; ADL: acid detergent lignin.

**Table 4:** Confusion matrix of post-assignment success per group (%) in the cross-validation.

	CAA	GLM	PLM	PMA
CAA	81.1	6.7	7.5	4.7
GLM	8.0	52.2	30.7	9.1
PLM	4.1	37.2	53.8	4.9
PMA	1.0	6.8	6.8	85.4

CAA: pure alfalfa crops; GLM: grass–legume mixtures recently established; PLM: permanent meadows originated from old grass–legume mixtures, PMA: permanent meadows originated from old alfalfa. Rows: actual classes to which samples belonged. Columns: classes to which samples were assigned.

**Table 5:** Confusion matrix of posterior assignment probability of each group in the semi-external validation.

	CAA	GLM	PLM	PMA
CAA	-	56.6	21.7	21.7
GLM	9.7	-	77.4	12.9
PLM	3.1	9.8	-	3.1
PMA	8.8	38.6	52.6	-

CAA: pure alfalfa crops; GLM: grass–legume mixtures recently established; PLM: permanent meadows originated from old grass–legume mixtures, PMA: permanent meadows originated from old alfalfa. Rows: actual classes to which samples belonged. Columns: classes to which samples were assigned.

## Data Availability

None of the data were deposited in an official repository. The datasets used and/or analyzed during the current study are available from the corresponding author upon reasonable request.

## Supplementary Materials

The following supporting information can be downloaded at: https://www.mdpi.com/article/10.3390/ani14182646/s1, Figure S1: Biplot of principal components analysis, where CAA is pure alfalfa crops; GLM is grass–legume mixtures; PLM is permanent meadows originated from old grass–legume mixtures and PMA is permanent meadows originated from old alfalfa.

## References

[1] Puche N., Senapati N., Flechard C.R., Klumpp K., Kirschbaum M.U.F., Chabbi A. (2019). Modeling Carbon and Water Fluxes of Managed Grasslands: Comparing Flux Variability and Net Carbon Budgets between Grazed and Mowed Systems. Agronomy.

[2] Dondini M., Martini M., De Camillis C., Uwizeye A., Soussana J.F., Robinson T., Steinfeld H. (2023). Global Assessment of Soil Carbon in Grasslands—From Current Stock Estimates to Sequestration Potential.

[3] Egoh B.N., Bengtsson J., Lindborg R., Bullock J.M., Dixon A.P., Rouget M. (2016). The Importance of Grasslands in Providing Ecosystem Services: Opportunities for Poverty Alleviation. Routledge Handbook of Ecosystem Services.

[4] Allan E., Bossdorf O., Dormann C.F., Prati D., Gossner M.M., Tscharntke T., Blüthgen N., Bellach M., Birkhofer K., Boch S. (2014). Interannual Variation in Land-Use Intensity Enhances Grassland Multidiversity. Proc. Natl. Acad. Sci. USA.

[5] Gossner M.M., Lewinsohn T.M., Kahl T., Grassein F., Boch S., Prati D., Birkhofer K., Renner S.C., Sikorski J., Wubet T. (2016). Land-Use Intensification Causes Multitrophic Homogenization of Grassland Communities. Nature.

[6] Suzuki Y., Okamoto H., Takahashi M., Kataoka T., Shibata Y. (2012). Mapping the Spatial Distribution of Botanical Composition and Herbage Mass in Pastures Using Hyperspectral Imaging. Grassl. Sci..

[7] Okamoto H., Murata T., Kataoka T., Hata S.-I. (2007). Plant Classification for Weed Detection Using Hyperspectral Imaging with Wavelet Analysis. Weed Biol. Manag..

[8] Ali I., Cawkwell F., Dwyer E., Barrett B., Green S. (2016). Satellite Remote Sensing of Grasslands: From Observation to Management—A Review. J. Plant Ecol..

[9] Fang P., Zhang X., Wei P., Wang Y., Zhang H., Liu F., Zhao J. (2020). The Classification Performance and Mechanism of Machine Learning Algorithms in Winter Wheat Mapping Using Sentinel-2 10 m Resolution Imagery. Appl. Sci..

[10] Zhang X., Liu J., Qin Z., Qin F. (2019). Winter Wheat Identification by Integrating Spectral and Temporal Information Derived from Multi-Resolution Remote Sensing Data. J. Integr. Agric..

[11] Zhang X., Qiu F., Qin F. (2019). Identification and Mapping of Winter Wheat by Integrating Temporal Change Information and Kullback–Leibler Divergence. Int. J. Appl. Earth Obs. Geoinf..

[12] Zhao Y., Zhu W., Wei P., Fang P., Zhang X., Yan N., Liu W., Zhao H., Wu Q. (2022). Classification of Zambian Grasslands Using Random Forest Feature Importance Selection during the Optimal Phenological Period. Ecol. Indic..

[13] Dale L.M., Thewis A., Boudry C., Rotar I., Păcurar F.S., Abbas O., Dardenne P., Baeten V., Pfister J., Fernández Pierna J.A. (2013). Discrimination of Grassland Species and Their Classification in Botanical Families by Laboratory Scale NIR Hyperspectral Imaging: Preliminary Results. Talanta.

[14] Alomar D., Fuchslocher R., Cuevas J., Mardones R., Cuevas E. (2009). Prediction of the Composition of Fresh Pastures by Near Infrared Reflectance or Interactance-Reflectance Spectroscopy. Chil. J. Agric. Res..

[15] Reddersen B., Fricke T., Wachendorf M. (2013). Effects of Sample Preparation and Measurement Standardization on the NIRS Calibration Quality of Nitrogen, Ash and NDFom Content in Extensive Experimental Grassland Biomass. Anim. Feed Sci. Technol..

[16] Parrini S., Staglianò N., Bozzi R., Argenti G. (2022). Can Grassland Chemical Quality Be Quantified Using Transform Near-Infrared Spectroscopy?. Animals.

[17] Cougnon M., Van Waes C., Dardenne P., Baert J., Reheul D. (2014). Comparison of near Infrared Reflectance Spectroscopy Calibration Strategies for the Botanical Composition of Grass-Clover Mixtures. Grass Forage Sci..

[18] Britz R., Barta N., Schaumberger A., Klingler A., Bauer A., Pötsch E.M., Gronauer A., Motsch V. (2022). Spectral-Based Classification of Plant Species Groups and Functional Plant Parts in Managed Permanent Grassland. Remote Sens..

[19] Mikhailova E.A., Bryant R.B., Cherney D.J.R., Post C.J., Vassenev I.I. (2000). Botanical Composition, Soil and Forage Quality under Different Management Regimes in Russian Grasslands. Agric. Ecosyst. Environ..

[20] (2023). Official Methods of Analysis, 22nd Edition. https://www.aoac.org/official-methods-of-analysis/.

[21] Van Soest P.J., Robertson J.B., Lewis B.A. (1991). Methods for Dietary Fiber, Neutral Detergent Fiber, and Nonstarch Polysaccharides in Relation to Animal Nutrition. J. Dairy Sci..

[22] R Foundation for Statistical Computing R Core Team (2013). R: A Language and Environment for Statistical Computing. http://www.R-project.org/.

[23] Jombart T. (2008). Adegenet: A R Package for the Multivariate Analysis of Genetic Markers. Bioinformatics.

[24] Olivieri A. (2018). Introduction to Multivariate Calibration: A Practical Approach.

[25] Berauer B.J., Wilfahrt P.A., Reu B., Schuchardt M.A., Garcia-Franco N., Zistl-Schlingmann M., Dannenmann M., Kiese R., Kühnel A., Jentsch A. (2020). Predicting Forage Quality of Species-Rich Pasture Grasslands Using Vis-NIRS to Reveal Effects of Management Intensity and Climate Change. Agric. Ecosyst. Environ..

[26] Parrini S., Acciaioli A., Franci O., Pugliese C., Bozzi R. (2019). Near Infrared Spectroscopy Technology for Prediction of Chemical Composition of Natural Fresh Pastures. J. Appl. Anim. Res..

[27] Milberg P., Bergman K.-O., Glimskär A., Nilsson S., Tälle M. (2020). Site Factors Are More Important than Management for Indicator Species in Semi-Natural Grasslands in Southern Sweden. Plant Ecol..

[28] Deak A., Hall M.H., Sanderson M.A., Archibald D.D. (2007). Production and Nutritive Value of Grazed Simple and Complex Forage Mixtures. Agron. J..

[29] Li C., Peng F., Xue X., You Q., Lai C., Zhang W., Cheng Y. (2018). Productivity and Quality of Alpine Grassland Vary with Soil Water Availability under Experimental Warming. Front. Plant Sci..

[30] Xu L., Deng D.-H., Cai C.-B., Yang H.-W. (2011). Automatic Discrimination of the Geographical Origins of Milks by Excitation-Emission Fluorescence Spectrometry and Chemometrics. J. Autom. Methods Manag. Chem..

[31] Dal Prà A., Bozzi R., Parrini S., Immovilli A., Davolio R., Ruozzi F., Fabbri M.C. (2023). Discriminant Analysis as a Tool to Classify Farm Hay in Dairy Farms. PLoS ONE.

[32] Zicarelli F., Sarubbi F., Iommelli P., Grossi M., Lotito D., Lombardi P., Tudisco R., Infascelli F., Musco N. (2022). Nutritional Characterization of Hay Produced in Campania Region: Analysis by the near Infrared Spectroscopy (NIRS) Technology. Animals.

[33] White G.A., Smith L.A., Houdijk J.G.M., Homer D., Kyriazakis I., Wiseman J. (2015). Replacement of Soya Bean Meal with Peas and Faba Beans in Growing/Finishing Pig Diets: Effect on Performance, Carcass Composition and Nutrient Excretion. Anim. Feed Sci. Technol..

[34] Argenti G., Parrini S., Staglianò N., Bozzi R. (2021). Evolution of Production and Forage Quality in Sown Meadows of a Mountain Area inside Parmesan Cheese Consortium. Agron. Res..

[35] Waramit N., Moore K.J., Heggenstaller A.H. (2011). Composition of Native Warm-Season Grasses for Bioenergy Production in Response to Nitrogen Fertilization Rate and Harvest Date. Agron. J..

[36] Asner G.P. (1998). Biophysical and Biochemical Sources of Variability in Canopy Reflectance. Remote Sens. Environ..

[37] Kjeldahl K., Bro R. (2010). Some Common Misunderstandings in Chemometrics. J. Chemom..

[38] Mseddi K., Alghamdi A., Sharawy S., Ibrahim N. (2017). Screening of Weeds and Their Effect on Alfalfa (*Medicago sativa*). Indian J. Agric. Sci..

[39] Reiné R., Ascaso J., Barrantes O. (2020). Nutritional Quality of Plant Species in Pyrenean Hay Meadows of High Diversity. Agronomy.

[40] Pirolli P., Card S. (1999). Information Foraging. Psychol. Rev..

