# Supplementary figures and images for: Discriminant Analysis as a Tool to Classify Grasslands Based on Near-Infrared Spectra
Source: Animals (Basel). 2024 Sep 12;14(18):2646. doi: 10.3390/ani14182646 (PMC11429457; doi:10.3390/ani14182646)

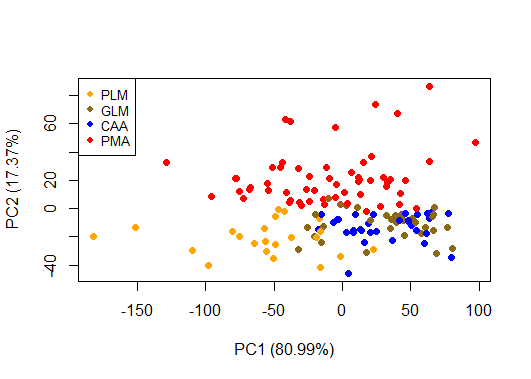

Supplement: Supplementary file 1 [file animals-14-02646-s001.zip › Figure S1.tiff]
